# Size‐Dependent Elastic Modulus and Core–Shell Structural Characteristics of Electrospun Nanofibers

**DOI:** 10.1002/mabi.202500280

**Published:** 2025-08-17

**Authors:** Muhammad Azeem Munawar, Fritjof Nilsson, Dirk W. Schubert

**Affiliations:** ^1^ Institute of Polymer Materials Department of Materials Science and Engineering Faculty of Engineering Friedrich–Alexander University Erlangen–Nürnberg (FAU) Martensstraße 7 91058 Erlangen Germany; ^2^ KeyLab Advanced Fiber Technology Bavarian Polymer Institute (BPI) Dr.‐Mack‐Straße 77 90762 Fürth Germany; ^3^ Division of Fibre and Polymer Technology School of Engineering Sciences in Chemistry, Biotechnology and Health KTH Royal Institute of Technology SE‐100 44 Stockholm Sweden; ^4^ FSCN – Fibre Science and Communication Network Mid Sweden University Sundsvall SE‐851 70 Sweden

**Keywords:** electrospinning, mechanical strength, nanofiber diameter, polycaprolactone (PCL), size‐dependent elasticity

## Abstract

This study investigates the size‐dependent mechanical properties of electrospun polycaprolactone (PCL) nanofibers by analyzing the relationship between fiber diameter and Young's modulus. Experimental data reveal a clear inverse trend: as fiber diameter decreases, stiffness increases significantly, indicating strong surface and confinement effects at the nanoscale. Two theoretical models were employed to interpret the observed behavior: a simplified core–shell model (Model 1) and an extended model (Model 2) incorporating surface tension and curvature elasticity. Both models accurately fit the experimental data across a diameter range of 450–850 nm, with Model 2 providing slightly better agreement at intermediate diameters (∼600–750 nm), where surface mechanics become more prominent. The enhanced stiffness in thinner fibers is attributed to increased surface‐to‐volume ratio and tighter molecular packing, while larger fibers exhibit bulk‐dominated mechanical responses. These findings highlight the importance of nanoscale geometry and surface effects in determining mechanical properties and suggest that fiber stiffness can be systematically tuned via diameter control during electrospinning.

## Graphical abstract

This figure illustrates the nanoscale mechanics of electrospun poly(ε‐caprolactone) (PCL) fibers, showing an inverse stiffness–diameter trend due to surface effects. A scanning electron microscopy (SEM) image depicts morphology, a modulus–diameter plot quantifies behavior, and two models—core–shell and surface‐curvature mechanics—reveal the shift from bulk to surface‐dominated mechanics via diameter tuning.

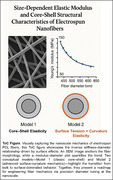

## 1 Introduction

Poly‐ε‐caprolactone (PCL) is a semicrystalline, biodegradable polyester that has attracted considerable attention over the past two decades due to its affordability, biocompatibility, and non‐toxic nature. These attributes make PCL highly suitable for a range of biomedical applications, including drug delivery systems, tissue engineering scaffolds, and long‐term implants. Its slow degradation rate and favorable mechanical properties further support its use in areas such as bone regeneration and resorbable sutures [1, 2, 3]. Among various fiber fabrication methods, electrospinning is the most widely employed technique for producing PCL nanofibers [4]. This is largely due to PCL's excellent spinnability, broad solvent compatibility, and ability to form uniform fibers with tunable structures under mild processing conditions [5, 6, 7]. Electrospun nanofibers possess an exceptionally high surface area‐to‐volume ratio, enhancing their functionality in diverse biomedical applications such as nerve regeneration [8], muscle tissue engineering [9], wound healing [10], cardiac tissue engineering [11], and bone regeneration [12]. These applications highlight the critical importance of tailoring nanofiber mechanical properties to meet specific functional requirements in biological environments [13].

The structural integrity and mechanical strength of PCL nanofibers are crucial for their effectiveness in tissue engineering [14]. Scaffold materials must exhibit sufficient mechanical robustness to support cell adhesion, proliferation, and migration while also closely mimicking the mechanical properties of native tissues [15]. This ensures structural integrity and provides mechanical support for tissue formation. Electrospinning parameters, including polymer solution concentration, molecular weight, and solvent composition, significantly influence fiber morphology and mechanical behavior [16]. Consequently, understanding the factors governing the mechanical properties of electrospun nanofibers is vital for optimizing their applications [17].

Numerous studies have demonstrated that the diameter of electrospun nanofibers significantly influences their mechanical properties, particularly the elastic modulus (E) and tensile strength. Higher E‐values are advantageous for applications demanding mechanical robustness and load‐bearing capacity, such as bone tissue engineering, vascular grafts, and ligament scaffolds. In contrast, lower E‐moduli are preferable for applications requiring flexibility and elasticity, including soft tissue engineering, skin regeneration, and wound dressings [18, 19, 20, 21, 22]. The diameter‐dependent variation in E is largely governed by electrospinning process parameters, such as polymer solution concentration, molecular weight, flow rate, and applied voltage [23]. In the present study, fiber diameter is controlled specifically by varying the polymer solution concentration. The observed size dependence of E is often attributed to increased molecular alignment and crystallinity within ultrafine fibers, resulting in enhanced mechanical performance [24, 25].

Additionally, advanced surface characterization techniques have revealed a distinct structural heterogeneity across electrospun fibers, often described by a core–shell morphology [24]. This structure originates from differential solvent evaporation rates during electrospinning, where the surface regions experience more rapid solvent loss compared to the fiber interior. The resultant anisotropic surface (shell) and more isotropic sub‐surface (core) regions can be effectively modeled as a two‐component core–shell system [26]. To rationalize the experimentally observed size dependence of the elastic modulus E as a function of fiber diameter D, a core–shell mechanical model is proposed. This model incorporates the composite nature of electrospun fibers, where the surface shell, being more oriented and crystalline, contributes disproportionately to the overall stiffness compared to the less‐ordered fiber core [24].

Building upon this concept, Subbotin et al. [27] recently introduced two theoretical models that predict the size dependence of the elastic modulus of electrospun nanofibers without relying on crystallinity changes. These models offer a novel framework for understanding and forecasting the mechanical behavior of electrospun fibers purely based on their diameter, paving the way for more precise control over their mechanical properties. However, further experimental validation and refinement of these models are necessary to fully grasp their implications in practical applications.

In this study, we systematically explored how fiber diameter influences the elastic modulus of highly oriented electrospun PCL nanofibers by adjusting the polymer solution concentration. Individual fiber mechanical properties were characterized using a commercial tensile testing system, enabling a direct correlation between diameter and mechanical performance. To further interpret the observed size‐dependent behavior, experimental data were evaluated against two core–shell mechanical models, offering insight into the structural heterogeneity inherent to electrospun fibers. This work advances the understanding of how nanoscale architectural features govern mechanical strength, providing a foundation for the rational design of electrospun scaffolds with tailored properties for targeted biomedical applications.

## 2 Materials and Methods

### 2.1 Materials

In this study, poly‐ε‐caprolactone (PCL) with a molecular weight (Mn) of 80 000 g/mol was obtained from Sigma–Aldrich (DE) and used as the base polymer for electrospinning. PCL was selected due to its well‐established biocompatibility, biodegradability, and suitability for biomedical applications. The solvents used for dissolving PCL were also sourced from Sigma–Aldrich (DE) and included chloroform (trichloromethane, TCM), dimethylformamide (DMF), and ethanol (EtOH).

### 2.2 Electrospinning of PCL

The electrospinning experiments were conducted using PCL, with the choice of solvents playing a critical role in determining fiber morphology, solution viscosity, and the mechanical properties of the electrospun nanofibers. A ternary solvent system consisting of TCM, DMF, and EtOH in a ratio of 4:0.3:0.7 was used to dissolve PCL. Chloroform served as the primary solvent due to its strong solubilizing power and ability to maintain favorable electrospinning conditions. DMF was added as a co‐solvent to regulate the solution's conductivity and influence fiber structure, as its high dielectric constant aids in fiber orientation. Ethanol was introduced to fine‐tune solvent volatility and evaporation rates, which in turn affected fiber diameter.

To prepare the electrospinning solutions, PCL was dissolved in the mixed solvent system under continuous magnetic stirring for 2 h at room temperature to ensure complete dissolution and a homogeneous polymer distribution. The PCL concentration was systematically varied between 10 and 22 g/mL to produce fibers with different diameters, enabling the investigation of size‐dependent mechanical properties. All chemicals were used as received without further purification to maintain consistency across experiments. The solvents were stored under controlled temperature and humidity conditions to minimize fluctuations in volatility and ensure reproducibility in fiber fabrication.

**FIGURE 1:**
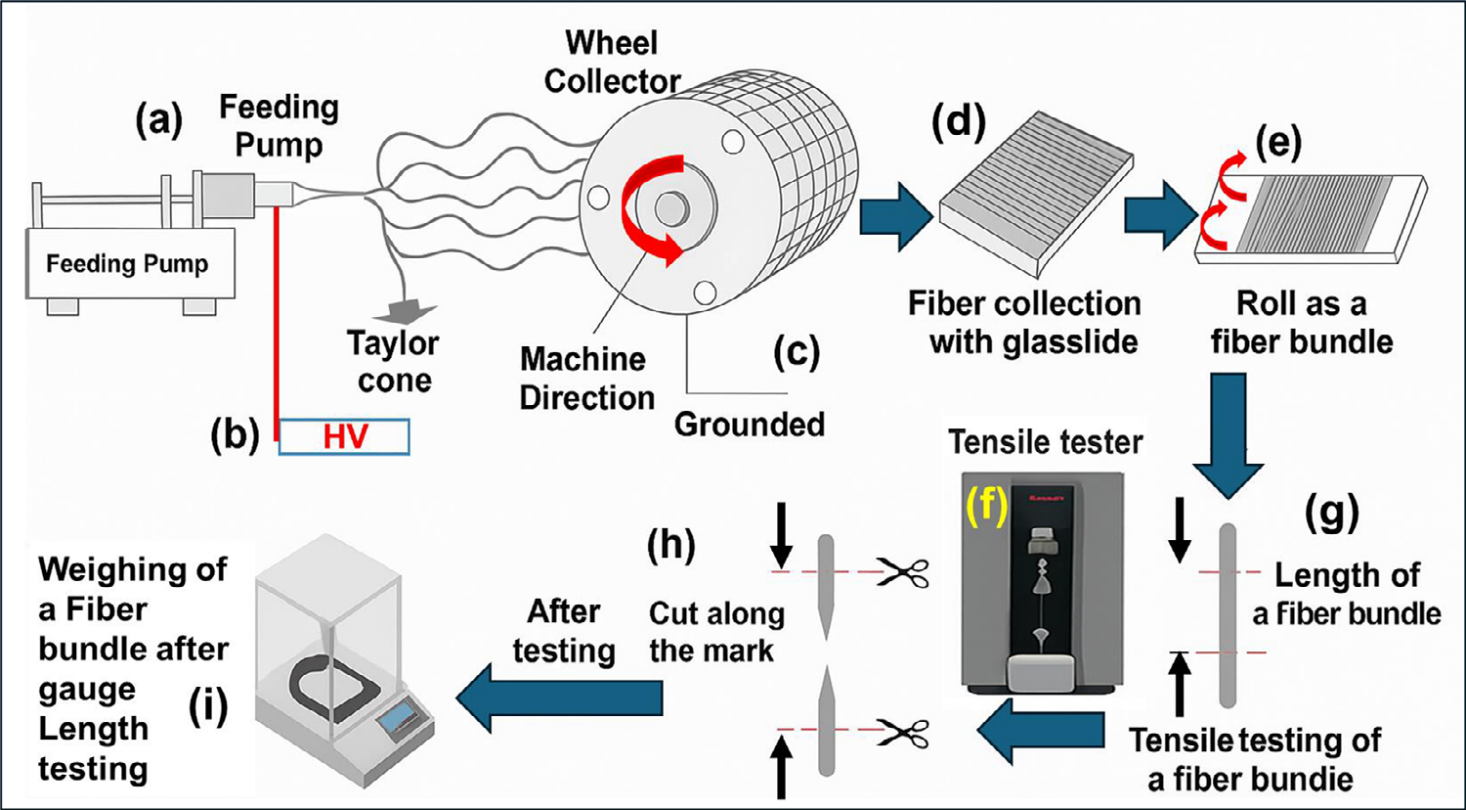
Schematic of electrospinning set‐up and procedure for mechanical properties measurement of oriented nanofiber bundles. (a–c) shows the feeding pump, high voltage power supply, and rotating wheel collector, respectively. The wheel covered with electrospun fibers is aligned with the machine direction. (d,e) shows the transfer and collection of fibers by the glass slide and the rolling of fibers into a bundle. (f–h) shows the tensile tester and mechanical properties measurement of fibers (within the red marks area). (i) shows the weighing of the tested sample within the marked length, respectively.

The prepared polymer solutions were then transferred into a 10 mL syringe with an internal cylinder diameter of 14.65 mm. An 18‐gauge needle was attached to the syringe, which was mounted on a syringe pump for controlled feeding. The electrospinning setup included a high‐voltage DC power supply (60 kV) from Linari Engineering S.r.l., Valpiana, Italy, which generated the required electric field to initiate fiber jet formation. Optimized electrospinning parameters are listed in Table 1. The entire fabrication process, sample preparation, and tensile testing workflow are illustrated in Figure 1.

**TABLE 1:** Optimized Parameters for Electrospinning PCL Solutions.

Feed rate (mL/h)	Voltage (kV)	Distance (cm)	Rotation rate (rpm)	Temperature (°C)	Humidity (%RH)
1.0	25	15	100	20–24	25–29

### 2.3 Specially Designed Rotating Wheel Collector for Fiber Alignment

A custom‐designed rotating wheel collector was employed to achieve highly aligned/oriented nanofiber deposition. The system featured a conductive cylindrical drum (21.2 cm in diameter) with two circular terminal plates mounted on either side. These plates supported multiple horizontal metal bars, which were evenly spaced and securely fixed using screws. During initial trials, some deformation of the fibers between the bars was observed, particularly in the early deposition layers. To address this, an optimization study was conducted to evaluate the effect of inter‐bar spacing on fiber alignment and structural integrity. It was determined that a spacing of 2.5 cm (25 mm) between adjacent bars provided the best balance‐minimizing sagging or distortion while maintaining a uniform and highly aligned fiber structure. This optimized configuration was used throughout the study to ensure consistent fiber orientation along the machine direction (MD).

**FIGURE 2:**
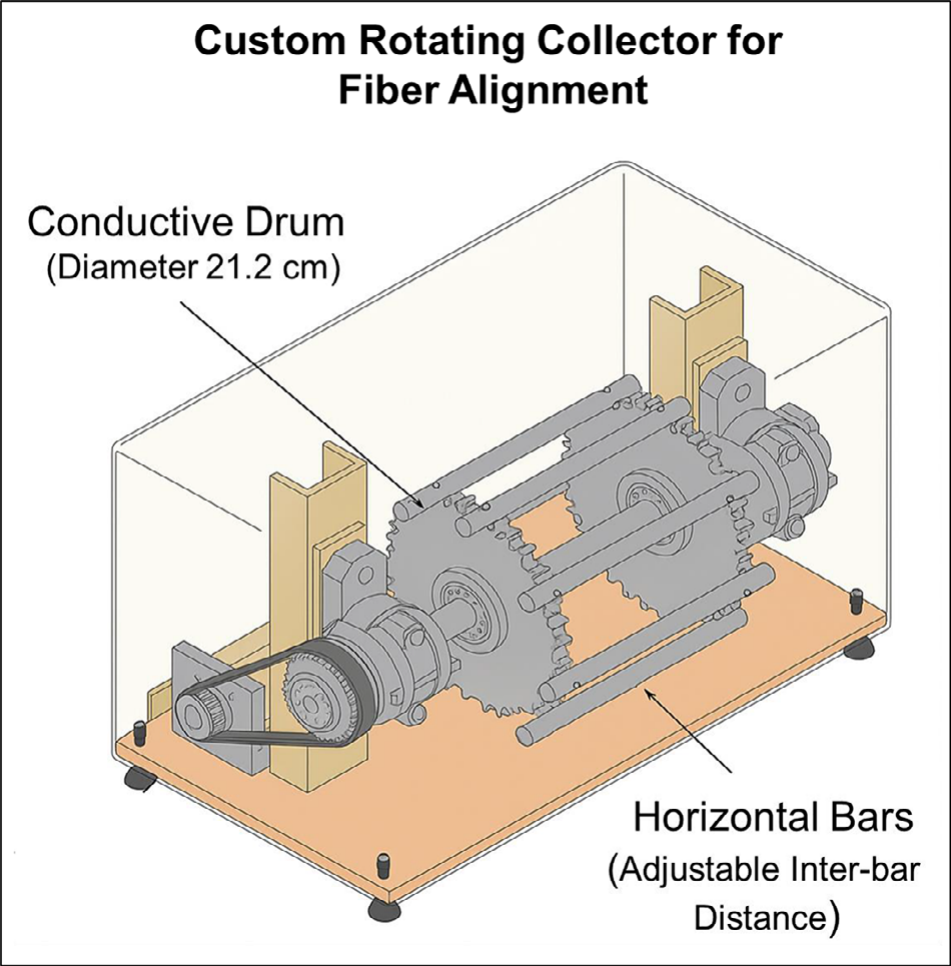
3D model of a rotating wheel collector featuring a conductive drum and horizontal bars for highly aligned nanofiber deposition during electrospinning.

During electrospinning, the cylinder rotated at a speed of 100 rpm, resulting in a tangential surface velocity of approximately 1.1 m/s. The applied electric field directed the PCL polymer jets toward the grounded collector. As the fibers approached the collector surface, the combination of the electrostatic attraction and the mechanical motion of the rotating drum stretched and deposited the fibers predominantly along the machine direction. The continuous rotation promoted a high degree of fiber orientation parallel to the rotation axis, enhancing the structural anisotropy critical for applications requiring aligned fibrous architectures. Figure 2 presents the 3D model of the custom‐designed rotating wheel collector used for aligned nanofiber deposition, illustrating the conductive drum and horizontal bar configuration.

### 2.4 Diameter Measurement of Electrospun Nanofibers

**FIGURE 3:**
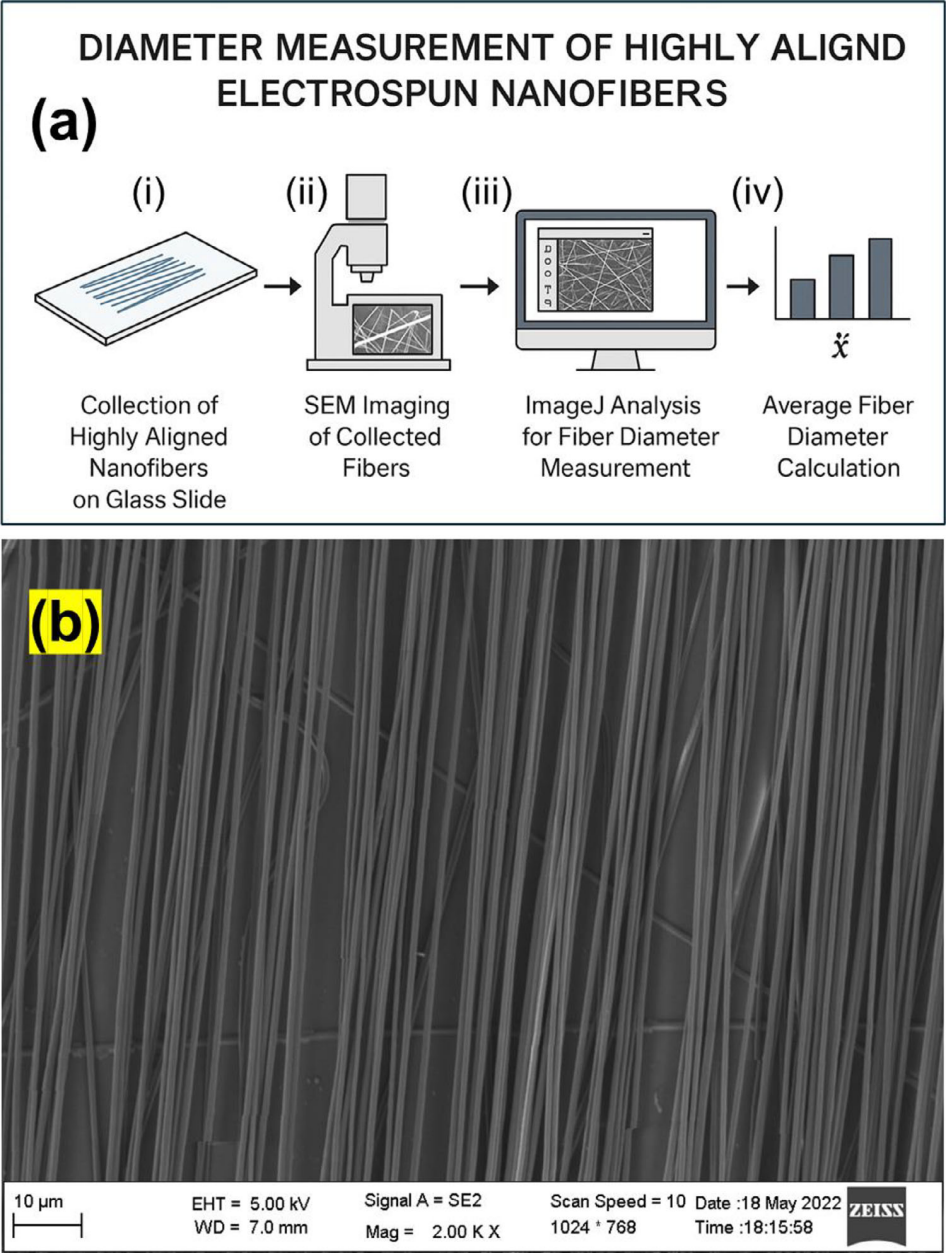
(a) Schematic illustration of the procedure for measuring the diameter of aligned electrospun nanofibers: (i) collection of aligned fibers on a glass slide, (ii) imaging of the fibers using SEM, (iii) image analysis using ImageJ software to measure individual fiber diameters, and (iv) calculation of the average fiber diameter from the measured data. (b) Representative SEM image showing the alignment of electrospun fibers on the glass substrate.

After electrospinning, the highly oriented nanofibers were carefully transferred from the rotating wheel collector onto glass slides. Special attention was given to preserve the alignment of the fibers during transfer, maintaining them as straight and parallel as possible between the bars of the rotating wheel to ensure reliable measurements. The average diameter of the PCL nanofibers was determined by analyzing scanning electron microscopy (SEM) images acquired using an AURIGA‐4750 microscope (ZEISS, Germany). Prior to SEM imaging, the glass slides with deposited fibers were trimmed into appropriately sized sections, mounted onto SEM stubs, and coated with a thin gold layer using a Q150T S sputter coater (Quantum Design Europe) under the WW5 Gold 1 program to minimize electron charging and improve image quality. SEM imaging was performed at magnifications of 2000× and 3000× to assess overall fiber morphology and distribution, and at 5000× to obtain accurate fiber diameter measurements. At each magnification, 50 different measurement points were randomly selected across the entire fiber matrix within the SEM images. Selection was performed manually without the use of a random number generator to ensure unbiased coverage of the fiber network. Fiber diameters were measured using ImageJ software. Careful random sampling across different regions of the fiber matrix enabled the acquisition of statistically significant and representative data. This approach provided an accurate and reproducible assessment of fiber size distributions, ensuring that the measured diameters reflected the actual effects of electrospinning parameters on the resulting nanofiber morphology. Figure 3 illustrates the methodology employed for measuring the diameter of aligned /oriented electrospun nanofibers. As shown in Figure 3a, the process involves sequential steps: collection of aligned fibers on a glass slide, imaging via SEM, and subsequent analysis using ImageJ software to obtain individual fiber diameters and calculate the average diameter. Figure 3b presents a representative SEM image demonstrating the uniform alignment of the electrospun fibers on the substrate, confirming the effectiveness of the alignment and collection method.

### 2.5 Mechanical Testing of Electrospun PCL Nanofibers

Aligned electrospun PCL fibers, transferred onto glass slides, were carefully rolled into bundles for tensile testing. To avoid fiber damage and preserve alignment, the rolling process was optimized using low, uniform pressure and controlled tension. Performing the procedure immediately after electrospinning leveraged residual tackiness, enhancing inter‐fiber cohesion and minimizing slippage or tearing.

**FIGURE 4:**
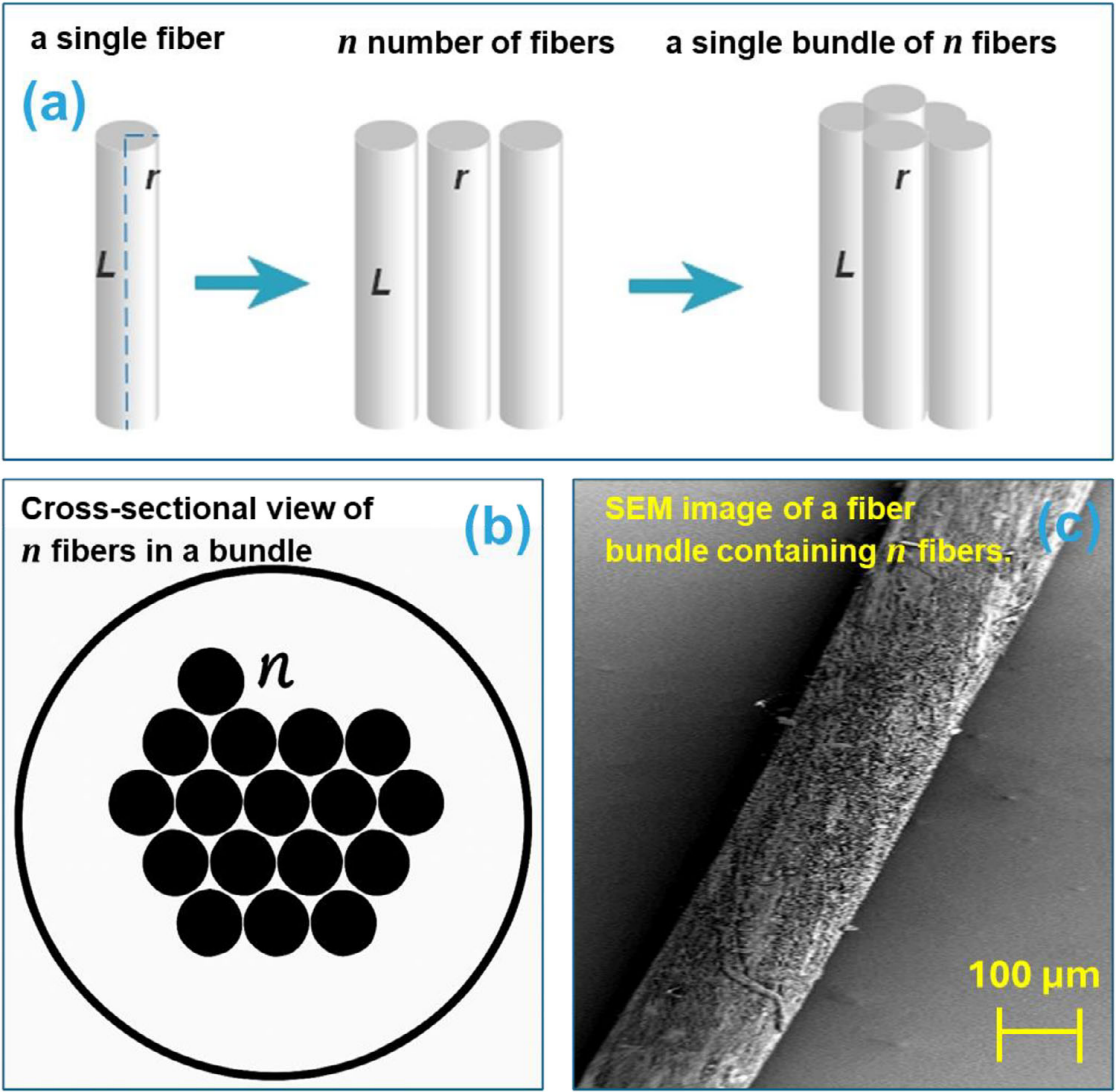
(a) Schematic of a single fiber (length L, radius r) and a bundle of n fibers for deriving the elastic Young's modulus (E) of nanofibers. (b) Schematic cross‐sectional view of n fibers in a bundle, illustrating the packing arrangement. (c) SEM image of a fiber bundle containing n fibers.

The mechanical characterization was based on the following assumptions: each nanofiber was considered a continuous, homogeneous, cylindrical structure, and the fibers within the bundle were tightly packed and aligned. As illustrated in Figure 4a, a schematic of a single fiber (length L, radius r) alongside a bundle of n fibers is used for deriving the elastic Young's modulus (E) of nanofibers. Figure 4b schematically illustrates the cross‐sectional view of n fibers within a bundle during packing, highlighting their arrangement and potential imperfections in the packing structure. Additionally, Figure 4c provides an SEM image of a fiber bundle containing n fibers. However, it is important to note that the actual cross‐sectional area of the fiber bundle A typically exceeds the cumulative sum of the cross‐sectional areas of the individual fibers (n ⋅ a), due to imperfect packing and void spaces between fibers. Therefore, A represents the effective cross‐sectional area of the bundle rather than the exact sum of fiber areas. This distinction is critical when interpreting the calculated mechanical properties.

**FIGURE 5:**
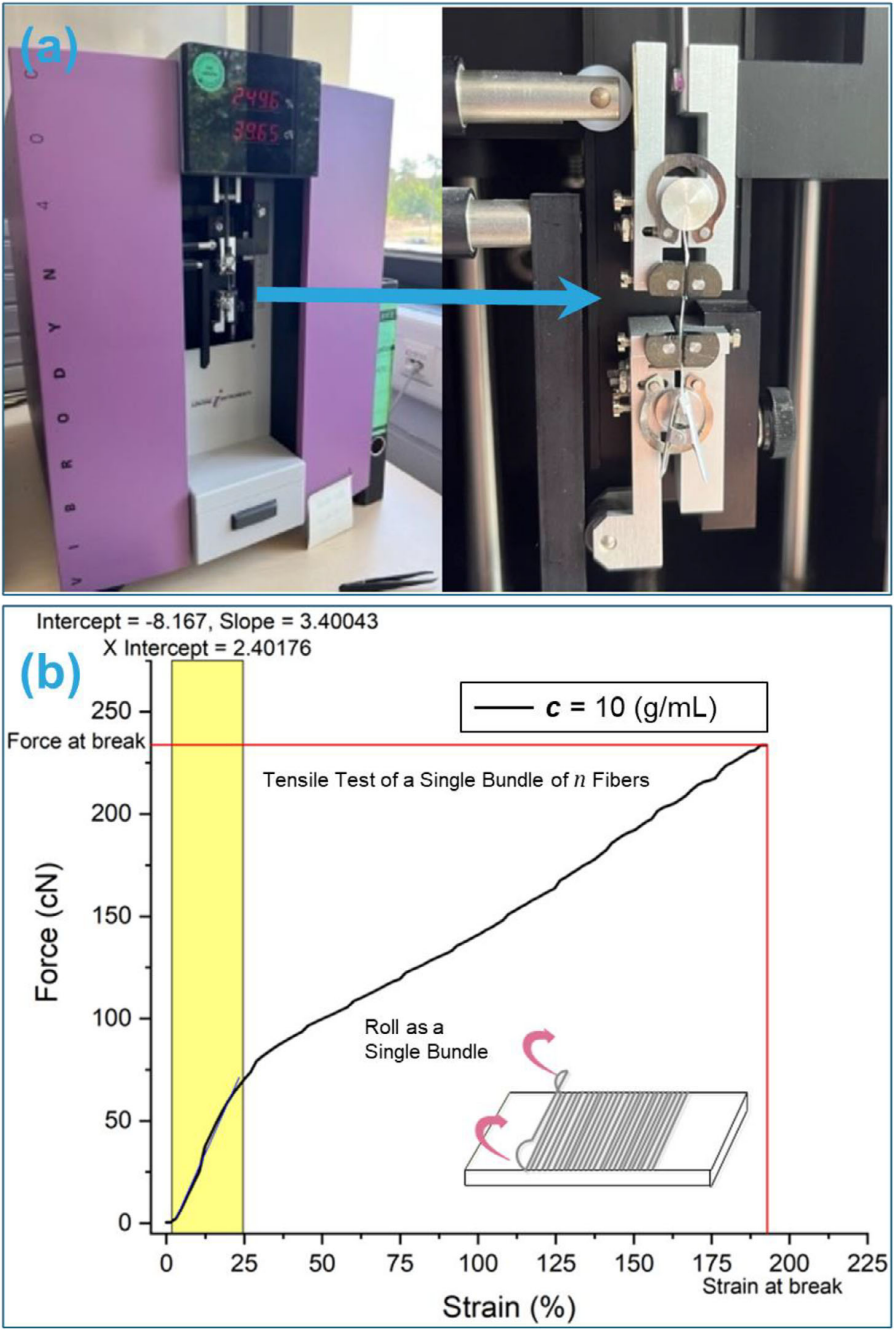
(a) Photograph of the Vibodyn 400 tensile tester (Lenzing Instruments GmbH & Co. KG, Gampern, Austria) used for tensile testing of nanofiber bundles. (b) A representative force‐strain curve for PCL nanofibers at a concentration of 10 g/mL, illustrating the determination of the slope k within the elastic deformation region up to 25% strain.

The **stress** (*σ*) and **strain** (*ϵ*) were determined by the standard definitions:

(1) $$\sigma =\frac{F}{A}$$

(2) $$\in =\frac{\Delta L}{L}$$

where *F* is the applied force, *A* is the effective cross‐sectional area of the fiber bundle, *ΔL* is the change in fiber length, and *L* is the initial gauge length. The tensile tester used to obtain the force‐strain curves is shown in Figure 5a. The representative force‐strain curve of PCL nanofibers at a concentration of 10 g/mL, presented in Figure 5b, illustrates the determination of the slope k within the elastic deformation region (up to 25% strain). The volume (V) of the fiber bundle was calculated using mass and density relations:

(3) $$V=\frac{W}{\rho }$$

(4) V=A·L

where W is the weight of the fiber bundle within the gauge length and ρ is the density of PCL material. By combining Equations (1)–(4), the Young's modulus (E) of an individual nanofiber can be derived using the following expression [28]:

(5) $$E=\frac{F\cdot L}{\Delta L\cdot A}$$

Substituting *A* from the volume equation gives:

(6) $$E=\frac{F\;\cdot L}{\Delta L\cdot \;\frac{W}{\rho \cdot L}\;}=\frac{F\;\cdot \rho \cdot \;L^{2}}{\Delta L\;\cdot \;W}\;$$

Further simplification leads to:

(7) $$E=k\cdot \frac{\rho \;\cdot L}{W}$$

where *k* is the slope of the force versus strain curve in the elastic region, defined as:

(8) $$k\;=\frac{F}{\in }\;=\frac{F\cdot L}{\Delta L}$$

It is crucial to differentiate between k and E: k represents the bundle stiffness per unit strain, which is influenced by the bundle geometry and fiber arrangement, whereas E is the intrinsic material property of the nanofibers, independent of geometric effects once they are taken into consideration. Force‐strain curves for the fiber bundles were obtained using a Vibodyn 400 tensile tester (Lenzing Instruments GmbH & Co. KG, Gampern, Austria) (Figure 5a). Mechanical testing was performed under controlled conditions using a preload of 100 mg, a gauge length of 5 mm, and an extension rate of 50 mm/min. Under these conditions, the electrospun PCL fiber bundles exhibited cohesive, uniform mechanical behavior, with no signs of progressive failure among individual fibers. Instead, the bundles responded as integrated structures, effectively behaving as single fibers with a unified cross‐sectional area. This consistent response is attributed to the high degree of fiber alignment and dense packing achieved during the collection and rolling process [29]. For reliability, three samples were tested for each fiber type. After tensile failure, the marked sections of each bundle were carefully collected, and their weight (W) was measured using a precision balance. The density (ρ) used in the calculations corresponds to the density of pure PCL material. The slope k was consistently determined from the force‐strain curves at 25% strain for all samples. Using the experimentally determined values of k, L, W, and ρ, the Young's modulus (E) of the individual nanofibers was then calculated using Equation (7). This approach offers a comprehensive framework for evaluating the elastic behavior of electrospun nanofibers and understanding the size‐dependent variation in mechanical properties, particularly the influence of fiber diameter on Young's modulus. Furthermore, this method is critical for accurately capturing the mechanical performance of nanofibers in biomedical applications such as scaffolds [28, 30].

## 3 Results and Discussion

### 3.1 Size‐Dependent Elastic Modulus and Mechanics of Electrospun Fibers

**FIGURE 6:**
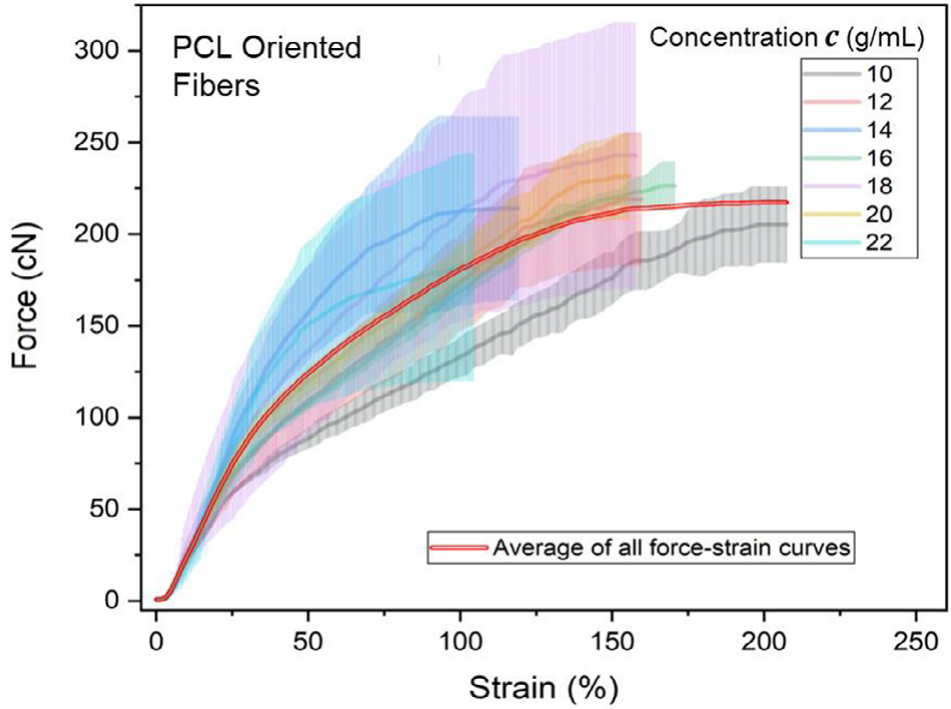
Force–strain curves of PCL nanofiber bundles fabricated from solutions with polymer concentrations ranging from 10 to 22 g/mL. Bold lines show the average response for each concentration, and the red double line represents the overall average. Shaded areas indicate variability between samples.

Figure 6 illustrates the force‐strain behavior of aligned/oriented electrospun polycaprolactone (PCL) fiber bundles fabricated from polymer solutions with concentrations ranging from 10 to 22 g/mL. The curves represent the averaged mechanical response from at least three independently tested samples per concentration. Each bold colored line denotes the average curve for a specific concentration, while the double bold red line shows the overall average behavior across all samples. The shaded regions convey the variability within each concentration group.

The mechanical response of the fiber bundles displays a clear dependency on polymer concentration. Electrospun fiber bundles produced from lower‐concentration solutions (10–16 g/mL) display a steep initial slope (k) within the elastic region (up to ∼25% strain), reflecting increased stiffness and resistance to deformation. In contrast, bundles from higher‐concentration solutions (18–22 g/mL) show more gradual slopes, reflecting increased compliance and a softer mechanical response. These variations are largely governed by the differences in solution viscosity, which influence jet dynamics during electrospinning. Lower‐viscosity solutions allow for greater elongational stretching and improved molecular alignment of polymer chains within the jet, which promotes better molecular organization and results in mechanically robust fibers [31, 32]. In contrast, high‐viscosity solutions limit jet stretching, leading to thicker fibers and reduced molecular alignment of polymer chains, thereby decreasing stiffness [19, 30, 33].

**FIGURE 7:**
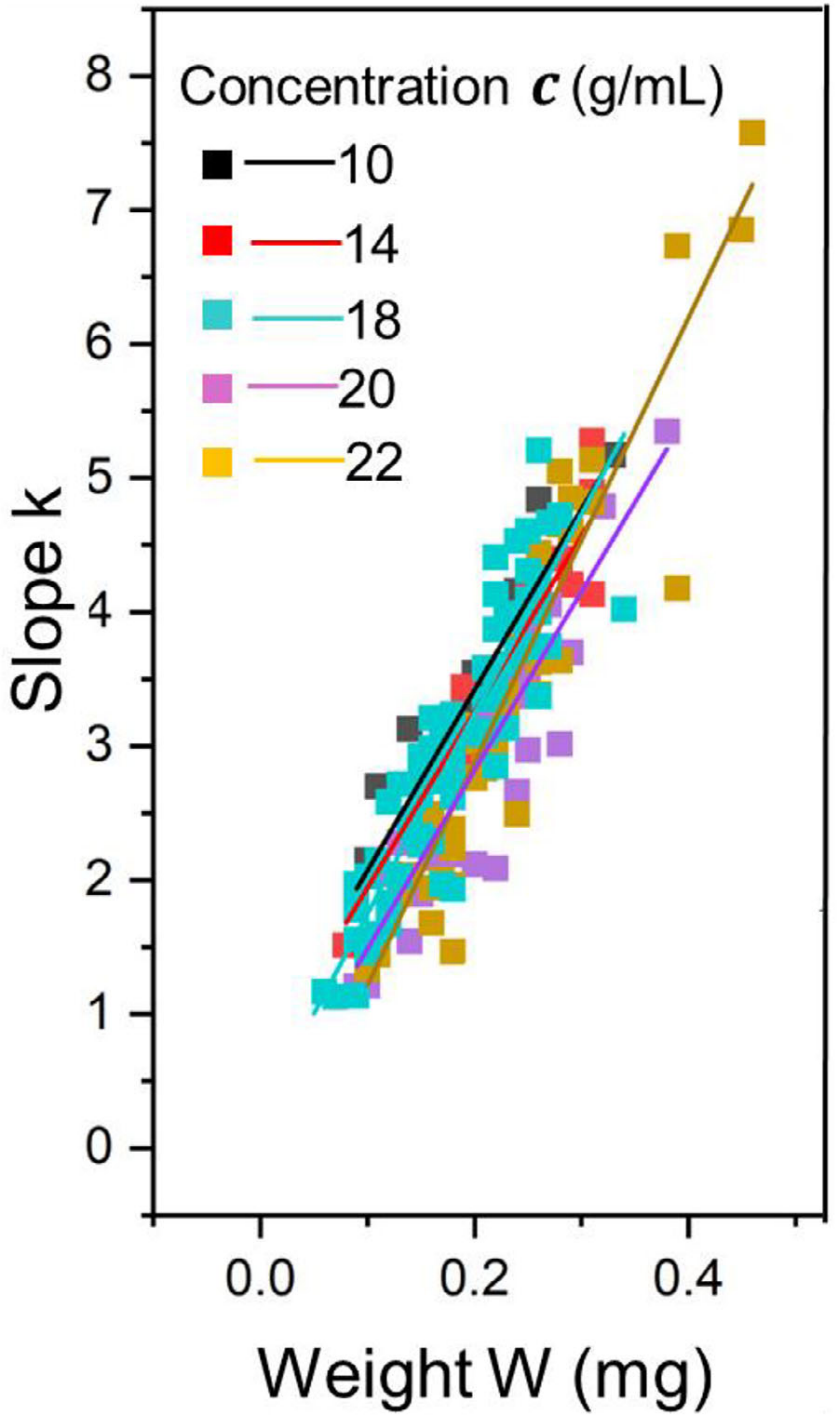
Plot of initial elastic slope (k) versus bundle weight (w) for electrospun PCL fiber bundles across selected polymer concentrations. Each data series corresponds to a specific concentration, with linear fits indicating a proportional increase in slope, reflecting a stiffer overall response of the fiber assembly. The results demonstrate a consistent linear relationship between k and w.

To explore how the mechanical response of fiber bundles scales with size, Figure 7 presents the initial slope (k) of the force‐strain curves as a function of bundle weight (W) across all concentrations. The analysis reveals a consistent trend: bundles with higher weights exhibit greater slope values, indicating a stiffer overall response of the fiber assembly. Distinct linear correlations within each concentration group suggest that increasing the number of fibers enhances load distribution and reduces inter‐fiber slippage due to tighter packing and better alignment.

However, as described by Equation (7), E = k⋅(ρ⋅L)/W, a higher bundle weight does not directly translate to a higher modulus of individual fibers. The increased slope reflects the collective mechanical behavior of the bundle rather than the intrinsic stiffness of single fibers. This distinction is important when interpreting the structural performance of the bundles. As further evidenced in Figure 7, the overall data points of the initial slopes for each concentration display linear fits, where fiber bundles from lower concentrations exhibit higher slopes. This reinforces the conclusion that fibers produced at lower concentrations possess higher intrinsic stiffness.

Notably, the highly aligned and compacted electrospun bundles responded as cohesive mechanical units with no signs of progressive individual fiber failure. This behavior supports the validity of weight‐based analysis in evaluating bundle‐level properties and highlights the structural integrity achieved through the optimized collection and rolling process [34, 35, 36].

**FIGURE 8:**
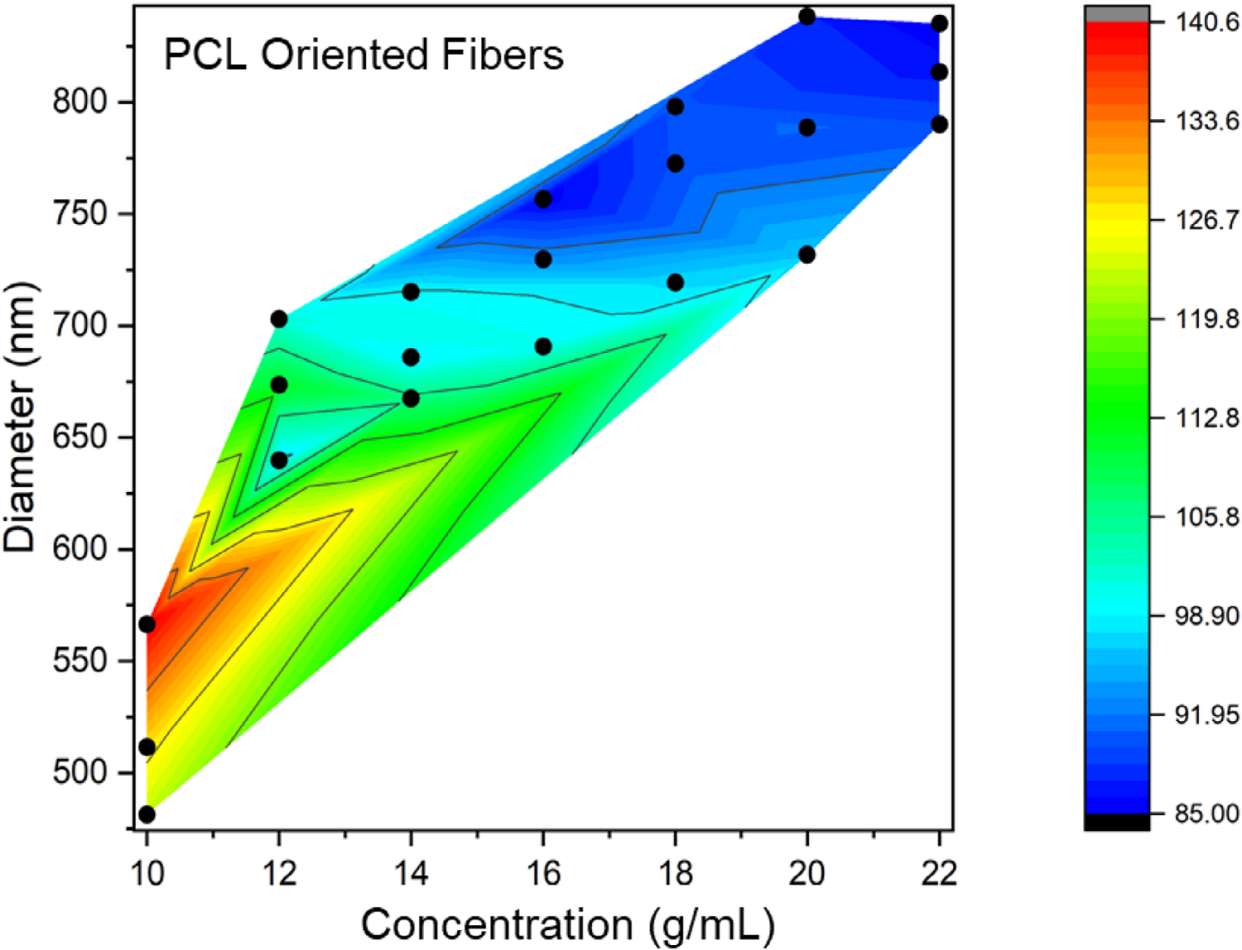
Contour plot illustrating the relationship between polymer concentration (g/mL), fiber diameter (nm), and Young's modulus (MPa) of electrospun PCL nanofibers. The plot demonstrates that increasing polymer concentration results in larger fiber diameters with reduced stiffness, while lower concentrations yield finer, stiffer fibers. The contour map was generated using Origin software with linear interpolation between experimentally measured data points; extrapolated regions are algorithmically estimated and should be interpreted qualitatively.

To quantify intrinsic stiffness, Young's modulus E was determined from the linear region of the force‐strain curves. The calculation accounted for the gauge length L, bundle weight W, and the density ρ of PCL. Further insight into the relationship between fiber morphology and mechanical properties is provided in Figure 8, which presents a contour plot mapping polymer concentration, fiber diameter, and Young's modulus. The *x*‐axis corresponds to polymer concentration (g/mL), the *y*‐axis to average fiber diameter (nm), and the color gradient represents Young's modulus (MPa). The plot demonstrates a coherent trend: fiber diameter increases with polymer concentration due to higher solution viscosity, while Young's modulus inversely decreases. This forms a triangular distribution‐thinner fibers produced from lower concentrations are mechanically stiffer (higher modulus), whereas thicker fibers formed from higher concentrations show reduced stiffness [18, 19, 37].

Together, Figures 6 through 8 present a comprehensive view of how polymer concentration affects fiber diameter, alignment, and stiffness. These findings establish that the mechanical performance of electrospun fibers is governed by an interplay between solution rheology, fiber morphology, and molecular architecture. By fine‐tuning the polymer concentration, it is possible to engineer fiber bundles with specific and predictable mechanical properties. This level of control is critical for applications requiring customized mechanical performance, such as tissue scaffolding, filtration systems, and soft robotics [22, 23, 25].

### 3.2 Core–Shell Model for Radius‐Dependent Young's Modulus

**FIGURE 9:**
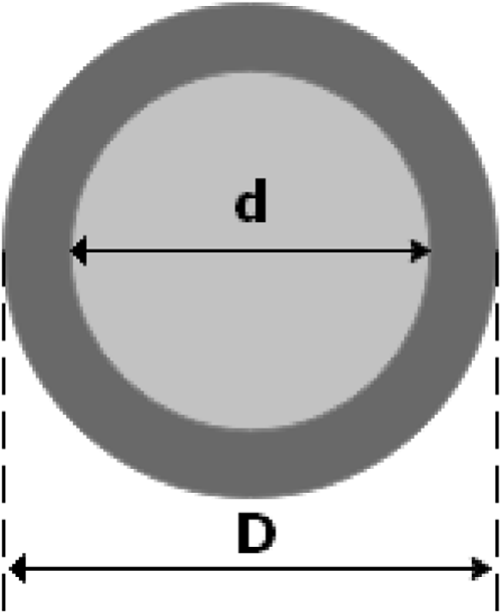
Schematic representation of a two‐component core–shell structure for an electrospun fiber, with d indicating the core diameter and D representing the total fiber diameter. The model illustrates the distinct morphological features of the core and shell regions developed during electrospinning.

During electrospinning, the polymer solution is subjected to a high electric field, causing the formation of ultrafine fibers as the solvent rapidly evaporates [38]. Due to faster solvent evaporation at the fiber surface compared to the core, a structural gradient develops within the fibers. Specifically, the surface region undergoes rapid solidification, leading to higher molecular alignment and crystallinity, while the core solidifies more slowly, maintaining a more isotropic and amorphous structure. This anisotropic internal configuration can be effectively modeled as a two‐component system: a highly oriented stiff shell surrounding a softer, less ordered core [39, 40, 41]. This structural configuration is effectively represented by a two‐component core–shell model, illustrated in Figure 9.

Such a core–shell morphology has been reported for several electrospun polymer systems [24, 39, 41], and its presence explains the experimentally observed size‐dependent mechanical properties in nanofibers, including polycaprolactone (PCL) fibers. Smaller diameter fibers tend to have a greater proportion of the stiffer shell relative to the core, resulting in higher overall mechanical stiffness. Therefore, understanding and modeling this structure is critical to accurately predicting the elastic behavior of electrospun nanofibers.

To predict the variation of Young's modulus with fiber diameter, two core–shell models were employed. Both models assume that the nanofiber consists of a homogeneous cylindrical core with elastic modulus E_c_, surrounding a shell with elastic modulus *E_S_*, and shell thickness δ, defined as δ = 0.5(D−d), where D is the total fiber diameter and d is the core diameter. It is important to note that *E_S_*, *E_C_* and δ are assumed to be independent of fiber radius for modeling simplicity. While a continuous gradient likely exists, the discrete two‐phase approximation provides a reasonable first‐order model that captures the essential mechanical behavior without introducing excessive complexity [42].

#### 3.2.1 Model 1: Core–Shell Model Based on Simple Mechanics

Following the approach derived by Subbotin et al. [27], and assuming uniform axial stress distribution under uniaxial loading, the effective Young's modulus E of the fiber can be expressed as:

(9) $$E=E_{C}+\left(E_{S}-E_{c}\right)\frac{4\delta }{D}$$

This expression was developed based on basic force balance and strain compatibility arguments across the core and shell regions. While sometimes referred to as “Hookean” behavior, it should be clarified that this model is not directly derived from Hooke's Law but from mechanical equilibrium considerations under elastic deformation. Equation (9) predicts that the effective Young's modulus increases as the fiber diameter decreases, due to the increasing contribution of the stiffer shell region.

#### 3.2.2 Model 2: Core–Shell Model Incorporating Surface and Curvature Effects

Recognizing that surface energy and curvature elasticity become significant at small scales, an extended model was proposed by Subbotin et al. [27], incorporating additional surface stress and bending stiffness contributions. In this model, the effective Young's modulus is expressed as:

(10) $$E=E_{c}+\frac{\left(\mu -\gamma \right)}{2r}+\frac{3K}{4r^{3}}$$

where: r is the fiber radius (r = D/2), µ represents the elastic modulus of surface stretching, γ is the surface tension, and K accounts for curvature‐dependent elastic effects.

Rearranging Equation (10) leads to a simplified form:

(11) $$E=E_{c}+\frac{K_{1}}{D}+\frac{K_{2}}{D^{3}}$$

where K_1_ = (µ—γ) is primarily influenced by the surface stretching modulus, and K_2_ = 6K is related to the curvature elastic modulus. Thus, Model 2 accounts for the size dependence of mechanical properties not only through core–shell morphology but also through explicit surface energy and curvature effects, which are especially important for submicron fibers. To ensure clarity and avoid ambiguity, the dimensional consistency of Equations (10) and (11) is verified. All terms in both equations are expressed in units of pressure (Pa), in agreement with the physical dimension of Young's modulus. In Equation (10), the surface term (µ−γ)/2r combines surface elastic modulus (µ, N/m) and surface tension (γ, N/m), divided by the radius (r, m), resulting in units of N/m^2^ (Pa). The curvature contribution 3K/4r^3^, with K in N·m, also yields N/m^2^ when divided by r^3^. In Equation (11), the terms K_1_/D and K_2_/D^3^ are likewise dimensionally consistent, with K_1_ in N/m and K_2_ in N·m. Each term thus retains compatibility with the units of Young's modulus, ensuring consistency within the model formulations.

#### 3.2.3 Fitting of Experimental Data Using the Core–Shell Model

**FIGURE 10:**
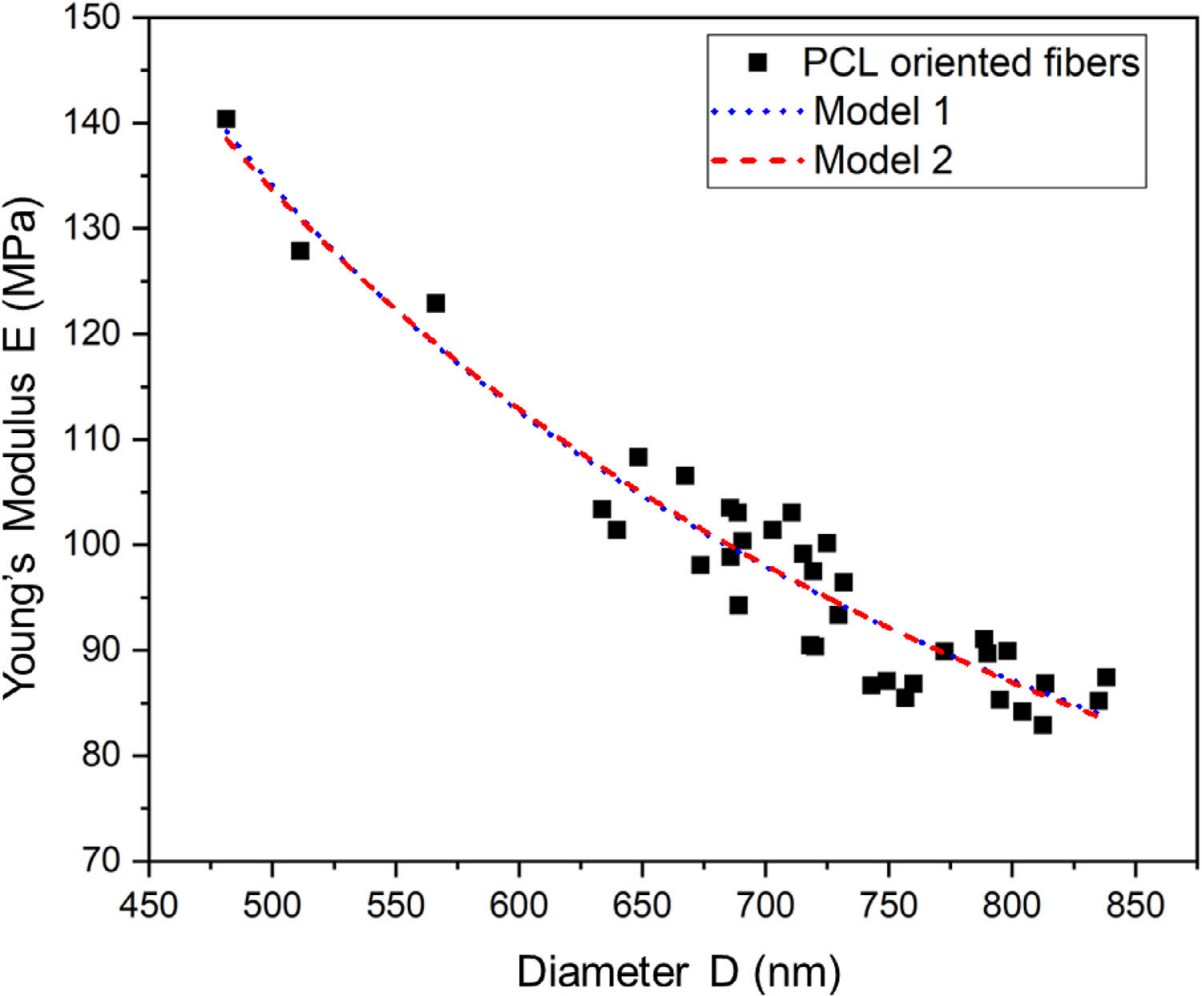
Fitting of experimental Young's modulus data for electrospun oriented fibers using two models: Model 1 (blue dots) based on core–shell mechanics and Model 2 (red dashed lines) incorporating surface tension and curvature effects. Both models show good agreement with the experimental data, confirming the core–shell structure and size‐dependent elasticity behavior.

To evaluate the mechanical behavior of electrospun PCL fibers and assess the validity of core–shell models, the experimental Young's modulus data were analyzed using two theoretical frameworks: Model 1, a simplified core–shell model, and Model 2, an extended model incorporating surface‐related effects such as surface tension and curvature elasticity. As shown in Figure 10, both models align well with the experimental results. Model 1 is represented by a blue dotted line, while Model 2 is denoted by a red dashed line. The associated fitting parameters for each model are presented in Table 2.

**TABLE 2:** Value of the parameters used for fitting of models.

Model	Ec (MPa)	4δ(Es‐Ec) × 10^−3^ (nm·MPa)	K_1_ × 10^−3^ (nm·MPa)	K_2_·× 10^−9^ (nm^3^·MPa)
1	9.06	62.30	—	—
2	15.67	—	55.86	0.84

A clear inverse relationship is observed between fiber diameter and stiffness. As the fiber diameter decreases, the Young's modulus increases significantly. Specifically, thinner fibers with diameters below ∼600 nm demonstrate enhanced stiffness (∼130–140 MPa), while thicker fibers above ∼750 nm exhibit lower stiffness (∼80–90 MPa). This size‐dependent mechanical strengthening can be attributed to increased surface‐to‐volume ratio, tighter molecular packing, and higher chain alignment in smaller fibers. These observations support the notion that surface effects become increasingly dominant at the nanoscale.

Both models successfully capture the diameter‐dependent trend in stiffness, confirming the applicability of a core–shell mechanical framework. Model 1 assumes a two‐phase structure with a softer core and stiffer shell and does not account for surface mechanics. It estimates a lower intrinsic core modulus (Ec = 9.06 MPa) and a substantial shell contribution (4δ(Es–Ec) = 62.3 × 10^−3^ nm·MPa). In contrast, Model 2 extends the framework by including surface tension (K_1_ = 55.86 × 10^−3^ nm·MPa) and curvature elasticity (K_2_ = 0.84 × 10^−9^ nm^3^·MPa), enabling a more accurate fit, particularly in the intermediate diameter range of 600–750 nm. The relatively small value of K_2_ suggests that curvature elasticity plays a secondary role within the 450–850 nm diameter range. Notably, Model 2 reduces to Model 1 when surface‐related parameters (γ and K) are set to zero, indicating that the models are fundamentally linked.

The improved performance of Model 2, especially at smaller diameters, highlights the growing influence of surface mechanics as fiber size decreases. Enhanced modulus in this regime is likely due to surface energy dominance and molecular confinement in the fiber shell. Conversely, in larger fibers, the bulk properties of the core dominate, leading to a reduction in overall stiffness. These findings underscore the critical role of surface effects in defining nanoscale mechanical behavior.

It is important to acknowledge the limitations of both models. The assumption that the core modulus (Ec), shell modulus (Es), and shell thickness (δ) are constant across all fiber sizes simplifies the modeling process but may not fully capture the gradual mechanical transitions occurring during fiber formation. In reality, a continuous property gradient likely exists due to solvent evaporation and relaxation dynamics during electrospinning. Despite this, the two‐phase approximation provides a reasonable and effective framework for describing mechanical properties within the studied diameter range.

In summary, the strong agreement between both models and the experimental data confirms that a core–shell structure, influenced by surface‐related phenomena, governs the radius‐dependent Young's modulus in electrospun fibers. These findings demonstrate that mechanical properties can be systematically tailored by controlling fiber size during fabrication. Future studies should focus on ultrathin fibers (<400 nm) to further validate the role of surface mechanics and refine predictive models for nanoscale systems.

## 4 Conclusions

The mechanical analysis of electrospun fibers demonstrates a strong inverse relationship between fiber diameter and Young's modulus, confirming that nanoscale dimensions significantly influence material stiffness. Thinner fibers (<600 nm) exhibit higher stiffness due to surface‐dominated mechanics, whereas thicker fibers (>750 nm) show reduced modulus consistent with bulk‐like behavior. Both theoretical models–Model 1 (core–shell) and Model 2 (extended surface mechanics)–successfully capture this trend, validating the core–shell framework for describing fiber elasticity. Model 2, which incorporates surface tension and curvature elasticity, offers enhanced predictive capability, particularly in the intermediate diameter regime.

The model fitting results underscore the relevance of nanoscale surface effects and suggest that the mechanical behavior of electrospun fibers can be tailored through diameter control. While the assumption of constant core and shell properties simplifies modeling, future work should explore gradient‐based approaches to better reflect the transitional nature of fiber formation. Investigations on ultrathin fibers (<400 nm) are especially needed to fully understand the impact of curvature and surface energy on mechanical performance. Overall, this study provides a foundation for engineering electrospun nanofibers with tunable mechanical properties for advanced applications in biomedical and structural materials.

## Conflicts of Interest

The authors declare no conflicts of interest.

## Data Availability

The data that support the findings of this study are available from the corresponding author upon reasonable request.
